# Short-term dexamethasone treatment transiently, but not permanently, attenuates fibrosis after acute-to-chronic kidney injury

**DOI:** 10.1186/s12882-018-1151-7

**Published:** 2018-12-03

**Authors:** Lies Moonen, Hilde Geryl, Patrick C. D’Haese, Benjamin A. Vervaet

**Affiliations:** Laboratory of Pathophysiology, University of Antwerp, 2160 Antwerpen, Belgium

**Keywords:** Renal fibrosis, Ischemia-reperfusion, Dexamethasone, Inflammation

## Abstract

**Background:**

Acute kidney injury (AKI) is an underestimated, yet important, risk factor for the development of chronic kidney disease (CKD). Persistence of inflammation after a renal ischemic injury has been observed, both in experimental models and patients, and is thought to be an important mechanisms underlying progression of acute-to-chronic renal injury. Temporary suppression of inflammation immediately after AKI might therefore be a good first-line therapeutic strategy towards a better long term outcome.

**Methods:**

Male C57Bl/6 J mice (Charles River, 10–12 weeks of age) underwent warm (36 °C body temperature) unilateral ischemia-reperfusion of the kidney for 21 min, after which treatment with intraperitoneal injection of the corticosteroid dexamethasone (10 mg/kg) was initiated for 3 weeks. Both at that time point and after an additional 3 week post-treatment follow up period, fibrosis was quantified by collagen I gene expression and immunostaining, as well as gene expression analysis of fibrosis-related genes *Tgfβ*, *Ccn2* (*Ctgf*), *Pai-1* and *Ccn3*. Furthermore, inflammation was evaluated by *Tnfα* gene expression and protein expression of the F4/80 macrophage marker and the α-SMA fibroblast marker. Lastly, renal histopathology was quantified by a morphometric analysis of the tubulointerstitial area.

**Results:**

Treatment with dexamethasone attenuated development of fibrosis, as evidenced by reduced collagen I gene expression and immunostaining, in combination with reduced gene expression of the pro-fibrotic *Ccn2* and increased expression of the anti-fibrotic *Ccn3*. The effects of dexamethasone on renal fibrosis persisted during the 3 week follow up period, as evidenced by stagnation of collagen I deposition in the ischemic kidney, in contrast to vehicle-treatment, where progression of fibrosis was observed. However, expression levels of the pro-fibrotic genes re-approached those of vehicle-treated injured kidneys suggesting that the effects of dexamethasone on fibrosis beyond the treatment period are temporary.

**Conclusion:**

A short term anti-inflammatory therapy with dexamethasone only transiently attenuates ischemia induced fibrosis. Prolonged or persistent anti-inflammatory treatment seems warranted to achieve long term benefit.

**Electronic supplementary material:**

The online version of this article (10.1186/s12882-018-1151-7) contains supplementary material, which is available to authorized users.

## Background

Acute kidney injury (AKI) is an underestimated, yet important, risk factor for the development of chronic kidney disease (CKD) [1]. Long-term follow-up studies (4 months to 6 years) report that between 35 and 71% of patients surviving an episode of AKI had incomplete recovery of renal function as assessed by creatinine clearance or serum creatinine measurements [2]. These patients are more likely to progress to end-stage renal disease (ESRD) as compared to patients without a history of either AKI or CKD [1] and contribute to the growing population of CKD patients. Currently, there are no therapeutic interventions targeting disease progression after AKI [3] highlighting the urgent need for novel therapeutic approaches that aim at preventing and/or reversing the pathophysiologic sequelae of AKI [4].

Renal ischemia-reperfusion injury, due to hypoperfusion after surgery, bleeding or dehydration, is a major aetiology in human AKI and is of particular importance in the setting of kidney transplantation [5]. We previously optimized a mouse model of AKI to CKD by unilateral ischemia-reperfusion (UIRI) without contralateral nephrectomy, with development of moderate renal fibrosis and significant long-term inflammation [6].

Inflammation plays a major role in the pathophysiology of ischemic AKI [7]. Post-ischemic tissue infiltration by neutrophils, macrophages, and different subtypes of T-cells is a hallmark of acute renal ischemic injury, both in patients and experimental models [7]. Persistence of inflammation may contribute to maladaptive cellular repair after acute injury and may be an intrinsic component of progression of renal injury [3]. In view of the above, attenuation of inflammation after acute ischemic kidney injury may be a suitable therapeutic strategy in the attenuation or prevention of progressive renal injury. Dexamethasone is a glucocorticoid, widely used in renal diseases as an anti-inflammatory and immunosuppressive agent [8]. Corticosteroids inhibit the synthesis of chemokines and cytokines resulting in protection against inflammation, and at high doses inhibit the immune response [9]. It was already shown in experimental models that dexamethasone has a protective effect against ischemic damage in liver and hart [10, 11]. In the kidney, pre-treatment with dexamethasone has been demonstrated to ameliorate the severity of an acute ischemic insult [8]. These studies, however, particularly covered the acute injury phase up to 24 h after the ischemic insult. In the light of the recently appreciated link between AKI and CKD, we here evaluated for the first time the long-term (up to 6 weeks) impact of temporary (3 weeks) inflammatory suppression by dexamethasone on pathology progression and development of fibrosis in ischemia-reperfusion injured kidneys.

## Methods

### Animals and experimental design

All animal procedures were conducted according to the National Institute of Health Guide for the Care and Use of Laboratory Animals and approved by the University of Antwerp Ethics Committee (approval number 2015–37).

Male C57Bl/6 J mice (10–12 weeks of age, Charles River, Saint-Germain-Nuelles, France) were randomly divided in 4 groups of which 3 (A, B and C) underwent 21 min of renal unilateral (left) ischemia-reperfusion injury (UIRI) at 36 °C core body temperature as described previously [6]. The fourth group (D) underwent Sham (i.e. mock UIRI) surgery. Groups were treated as follows: Group A) UIRI + no treatment (*n* = 8), Group B) UIRI + dexamethasone (*n* = 10, 10 mg/kg, intraperitoneal, daily for 3 days, then every other day as reported previously by Zager et al. 2011 [12]), Group C) UIRI + vehicle (*n* = 10, PBS, intraperitoneal, same volume of dexamethasone, daily for 3 days, then every other day), and Group D) Sham (i.e. mock surgery) + no treatment (*n* = 10). Treatment, via intraperitoneal injection, was initiated approximately 2 h after UIRI and lasted for 3 weeks. Animals of groups A and D, and half of the animals of group B and C were euthanized at the end of the 3-week treatment regimen. The other half of group B and C were euthanized at week 6, after an additional 3 weeks of follow-up without treatment. Prior to surgery, animals were randomly allocated to the different treatment groups. During the course of the study animals had free access to standard chow and tap water. On average we encountered 5% mortality, mainly due to post-anaesthetic complications. In addition, ca. 10% of the animals were excluded from analysis because their values were marked as outliers for multiple parameters upon statistical analysis. Removing outliers did not influence significances.

#### Surgical modalities

Renal unilateral ischemia-reperfusion injury (UIRI) was performed as described previously [6]. Animals were anesthetized with a combination of ketamine (Ketalar, Pfizer, Elsene, Belgium; 80 mg/kg) and xylazine (Rompun, Bayer, Wuppertal, Germany; 16 mg/kg) diluted in saline, via intraperitoneal injection. Buprenorphine (Reckitt Benckiser Healthcare, Brussel, Belgium; 0.05 mg/kg) was administered for pain relief. Immediately after loss of the righting reflex, the animals were placed on a heated surgical pad (Physitemp, Clifton, New Jersey), keeping body temperature of the animals at 36 °C. The abdomen was opened with a midline incision and the renal pedicle was carefully dissected and clamped with an atraumatic vascular clip (Scanlan, Saint Paul, Minnesota) for 21 min, continuously monitored with a rectal thermometer (Bioseb, Vitrolles, France). The right kidney was left undisturbed. To initiate reperfusion, the vascular clip was released and after verification of kidney colour change back to red, a Vicryl 4–0 suture (Ethicon, Norderstedt, Germany) was used to close the abdominal muscle layer and skin separately. Sham-operated animals received the same surgical procedure except placement of the clamp. Postoperatively, mice were given 1 ml saline intraperitoneally and were fed DietGel Recovery Purified Soft Diet for Rodents (Clear H_2_O, Norderstedt, Germany) for 3 days to reduce the post-operative weight loss and allow faster recovery after surgery.

#### Euthanasia

Animals were euthanized by exsanguination via the abdominal vena cava under ketamine-xylazine anaesthesia. Kidneys were excised, renal pole fractions were snap frozen in liquid nitrogen and transversal slices of renal tissue were fixed in methacarn (60% methanol, 30% chloroform, 10% acetic acid) and NBF (10% neutral buffered formalin) for 4 h and 24 h, resp., rinsed with 70% isopropanol and embedded in paraffin for histology.

### Real-time PCR

Total mRNA was extracted from a pole section of the ischemic kidney (PureLink RNA Mini Kit; Life Technologies, Gent, Belgium) and converted to cDNA (High Capacity cDNA archive kit; Life Technologies). qPCR, (ABI Prism 7000 sequence detection system; Life Technologies), was performed using Taqman probes and primers for *Gadph* (Mm99999915_g1), *collagen I α1* (Mm00801666_g1), *Tgfβ1* (Mm01178820_m1), *Ccn2* (Mm01192931_g1), *Ccn3* (Mm00456855_m1), *Pai-1* (Mm00435860_m1) and *Tnfα* (Mm00443258_m1). Each gene was analysed in triplicate and the expression was normalized to the reference gene *Gadph* Calculations were made conform the comparative Ct-method [13].

### Histology

Paraffin embedded 4 μm thick sections of ischemic kidney tissue were blocked with normal goat serum and incubated overnight with primary antibody, resp. polyclonal rabbit anti-mouse collagen I antibody (T40777R, Biodesign International, Saco, Maine) for evaluation of fibrosis, polyclonal rabbit anti-mouse Ki67 (Novus, Abingdon, UK) for evaluation of proliferation, polyclonal rabbit anti-mouse α-SMA (ab5694, Abcam, Cambridge, UK) for evaluation of activated fibroblasts, and polyclonal rabbit anti-mouse F4/80 (sc-25,830, Santa Cruz, Heidelberg, Germany) for evaluation of monocytes/macrophages. Subsequent incubation with a biotinylated goat anti-rabbit IgG antibody (PK-4001, Vector Laboratories, Burlingame, California), 3% H_2_O_2,_ avidin and biotinylated horseradish peroxidase (AB-complex, Vector Laboratories) allowed visual detection of a dark brown colour upon addition of diaminobenzidine. Sections were counterstained with methyl green to visualize nuclei. Additionally, paraffin embedded 4 μm thick sections of ischemic kidney tissue were stained with periodic acid-schiff stain (PAS) to quantify the tubulointerstitial area. Immunostainings were quantified with Axiovision image analysis software (Carl Zeiss, Jena, Germany) and ImageJ (FIJI) [14]. For collagen I and α-SMA quantification, digital photographs were taken (complete slide at 100x and 50x respectively). The area % positive stain represents the ratio of the summed absolute areas of staining versus the total tissue. Ki67 and F4/80 quantification was performed on 5 random cortical and outer medulla fields per kidney (500x). Data are presented as the mean number of Ki67 positive tubular nuclei per 5 fields (+/− SD) or as area % positive stain respectively. Assessment of renal pathology was performed on 3 random microscopic fields (400x) captured within the outer medulla on a PAS-stained section of the left kidney (1 section per animal). Tubulointerstitial area was quantified histomorphometrically using FIJI image analysis [14] by calculating the ratio between the number of pixels taken by the region that contains no intact tubules, no glomeruli, nor vessels and the number of pixels taken by the total picture, resulting in the area % of tubulointerstitium (+/− SD).

### Western blotting

Total protein was isolated from a pole section of the ischemic kidney using RIPA buffer, after which protein concentration is determined by the colorimetric, Pierce™ BCA protein assay kit (Fisher Scientific, Landsmeer, Netherlands). Proteins were separated through SDS-PAGE gel electrophoresis (35 min at 165 V) on 4–20% precast polyacrylamide gels (Bio-Rad, Nazareth, Belgium) and transferred onto a PVDF blotting membrane with 0.45 μm pore size (GE Healthcare Life Sciences, Diegem, Belgium) for 60 min at 100 V. The membrane was then incubated in 5% non-fat milk (blotting grade blocker, Biorad) for 2 h and subsequently overnight with the primary antibody, resp. polyclonal rabbit-anti-mouse β-actin (4970 S, Cell Signalling, Leiden, Netherlands), polyclonal rabbit-anti-mouse F4/80 (sc-25,830, Santa Cruz, Heidelberg, Germany) and rabbit-anti-mouse α-SMA (ab5694, Abcam, Cambridge, UK), diluted in 1% non-fat milk and subsequently incubated with a HRP conjugated goat-anti-rabbit IgG (sc-2004, Santa Cruz). The immune complexes were detected using a chemiluminiscence kit (SuperSignal West; Fisher Scientific).

### Statistics

All analysis were performed in a blinded manner. Statistical analysis was performed with SPSS Statistics 22 (IBM, Brussel, Belgium). Data are presented as mean ± standard deviation, or as individual values with median. Comparisons between groups are assessed using a Kruskal-Wallis test, followed by a Mann-Whitney U test. Values of *p* < 0.05 are considered significant. Identification of outliers was based on the internal method of SPPS for outlier detection build on the 1,5 x IQR (interquartile range) rule.

## Results

### Progression from AKI to CKD in untreated and vehicle-treated animals

**Fig. 1:**
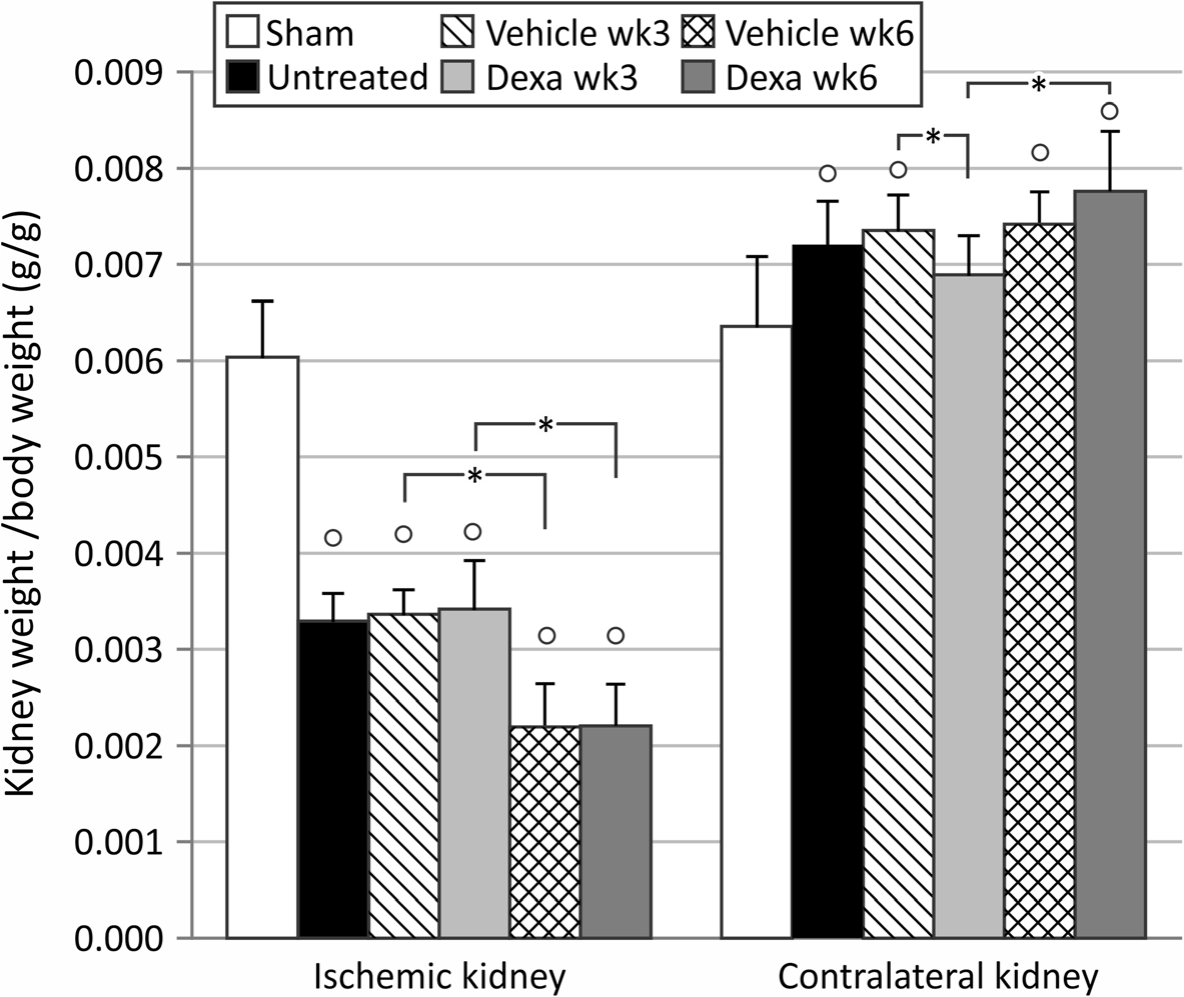
Mass of the ischemic and contralateral kidneys at euthanasia, corrected for body weight. UIRI was performed for 21 min at 36 °C, *n* = 8 in the untreated group, *n* = 10 in sham, dexamethasone and vehicle treatment groups. Animals were euthanized after treatment (3 weeks after UIRI) and 3 weeks after treatment (6 weeks after UIRI). °: *p* < 0.05 vs. Sham, *: *p* < 0.05

As depicted in Fig. 1, renal unilateral ischemia-reperfusion injury (UIRI) without contralateral nephrectomy induced a significant reduction of renal mass as compared to sham-operated animals (3.29 ± 0.29 mg/g vs. 6.04 ± 0.58 mg/g) (*p* < 0.05) 3 weeks after UIRI. The severity of the renal atrophy is equal in ischemic kidneys of vehicle-treated and untreated animals (Fig. 1). The weights of the contralateral uninjured kidney increased after UIRI in untreated animals as compared to sham (7.21 ± 0.45 mg/g vs. 6.36 ± 0.73 mg/g) (*p* < 0.05), which is in accordance with the physiological fact that the contralateral kidney becomes hypertrophic to compensate for the loss of function of the ischemic kidney (Fig. 1). The extent of hypertrophy is similar in vehicle-treated animals and untreated animals.

**Fig. 2:**
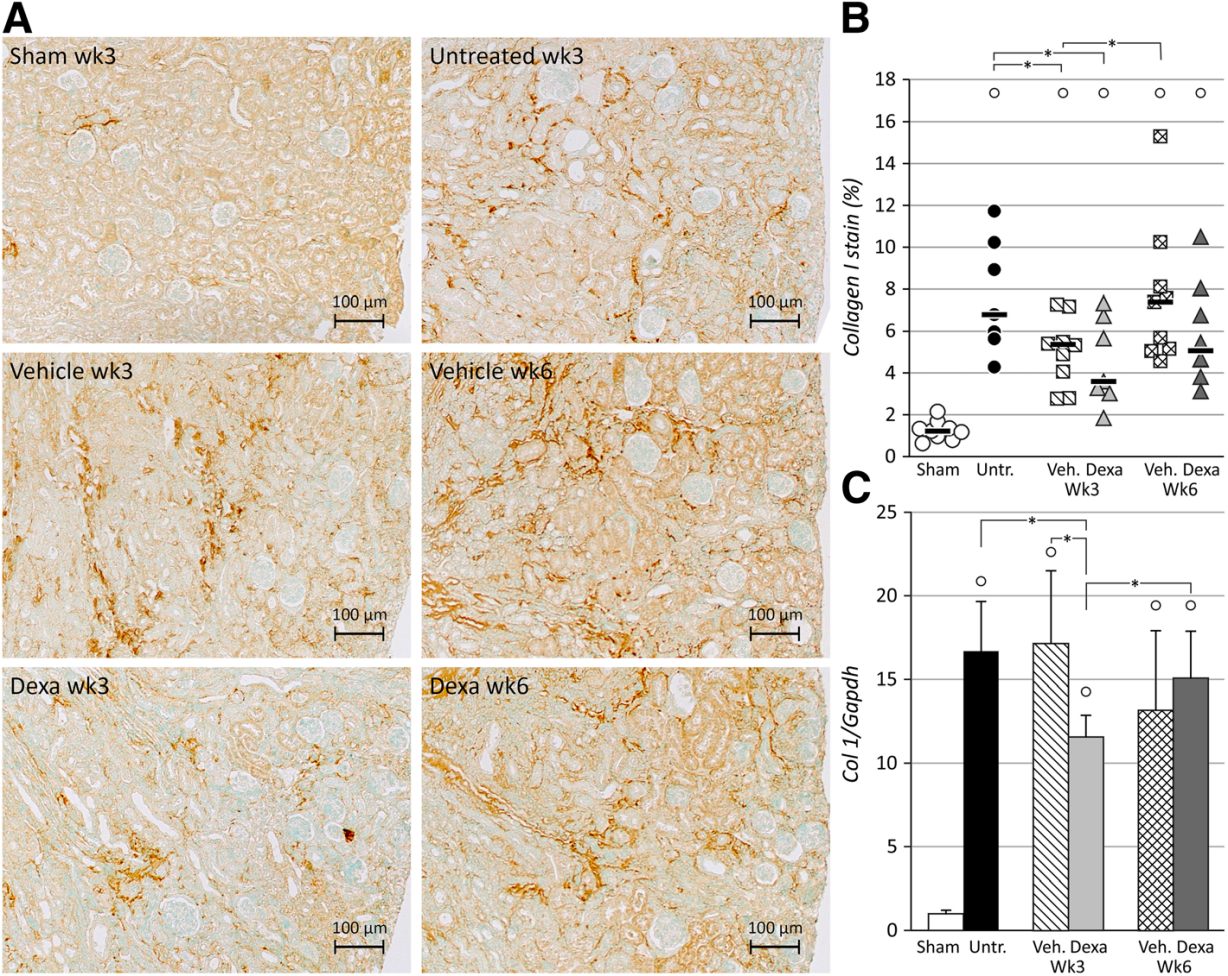
Evaluation of long-term fibrosis development in the ischemic kidneys. UIRI was performed for 21 min at 36 °C, *n* = 8 in the untreated group, *n* = 10 in sham, dexamethasone and vehicle treatment groups. Animals were euthanized after treatment (3 weeks after UIRI) and 3 weeks after treatment (6 weeks after UIRI). **a**: Representative images of collagen I immunostained ischemic kidney tissue (magnification: 100x). **b**: Histological quantification of collagen I protein in ischemic kidneys (magnification: 50x). **c**: *collagen I* gene expression (qPCR). °: *p* < 0.05 vs. Sham, *: *p* < 0.05

**Fig. 3:**
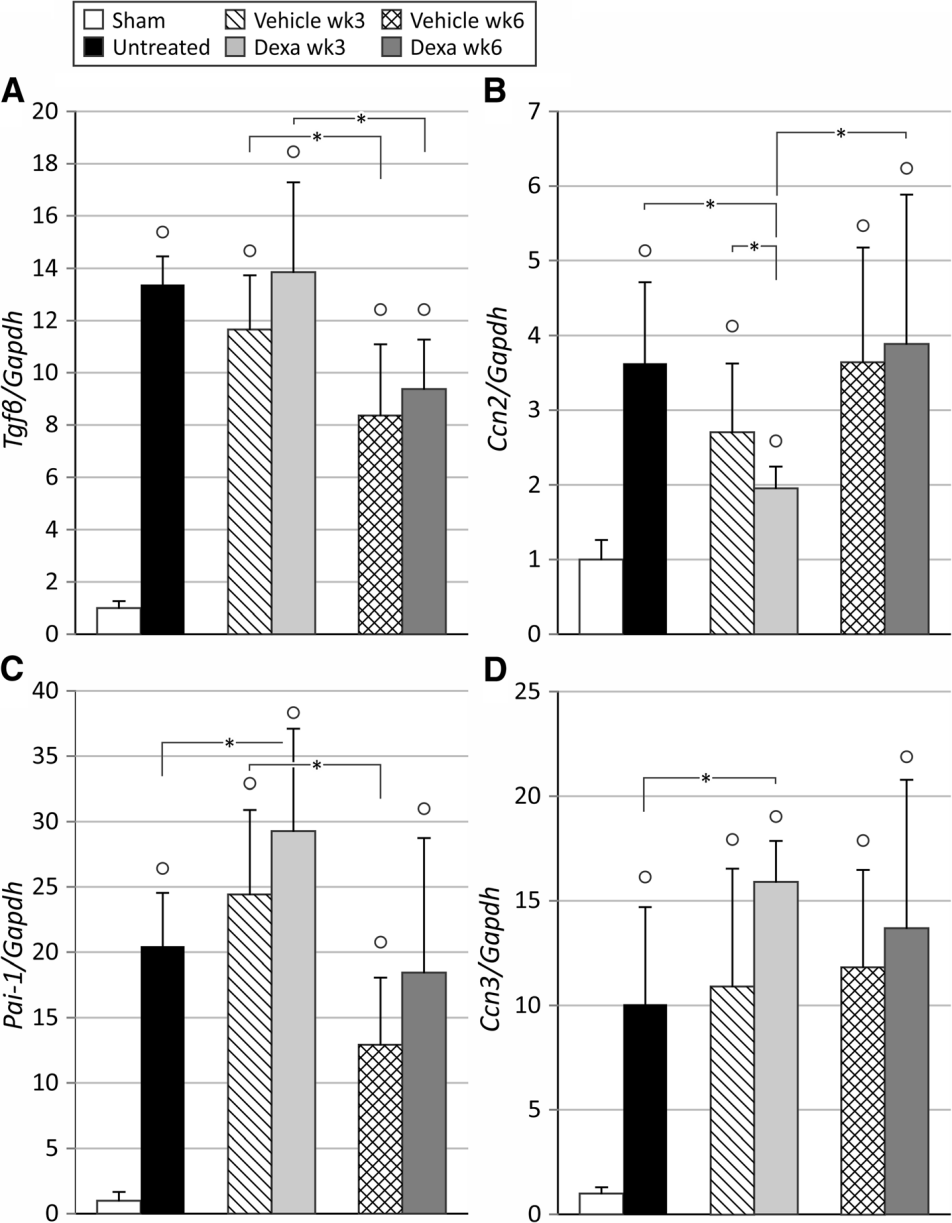
Analysis of expression of fibrosis-related genes in the ischemic kidney. UIRI was performed for 21 min at 36 °C, *n* = 8 in the untreated group, *n* = 10 in sham, dexamethasone and vehicle treatment groups. Animals were euthanized after treatment (3 weeks after UIRI) and 3 weeks after treatment (6 weeks after UIRI). **a**: *Tgfβ* gene expression (qPCR). **b**: *Ccn2* (Ctgf) gene-expression (qPCR). **c**: *Pai-1* gene expression (qPCR). **d**: *Ccn3* gene expression (qPCR). °: *p* < 0.05 vs. Sham, *: *p* < 0.05

Significant upregulation of expression of fibrosis-related genes *collagen I* (16.7 ± 3.0 fold), *Tgfβ* (13.4 ± 1.1 fold), *Ccn2* (*Ctgf*) (3.6 ± 1.1 fold), *Ccn3* (10.1 ± 4.6 fold), *Pai-1* (20.5 ± 4.1 fold) and *Tfnα* (11.3 ± 4.8 fold) was observed in the ischemic kidneys of untreated animals as compared to shams (*p* < 0.05) 3 weeks after UIRI (Fig. 2c and Fig. 3a-d). Vehicle-treatment did not significantly influence this expression pattern. In addition, significantly more collagen I deposition (i.e. fibrosis) was present in the kidneys of untreated UIRI animals as compared to shams (7.6 ± 2.7% vs. 1.3 ± 0.5%) (*p* < 0.05) (Fig. 2). After vehicle treatment, this effect was maintained, although to a somewhat lesser extent (5.1 ± 1.5% vs. 7.6 ± 2.7%) (*p* < 0.05).

**Fig. 4:**
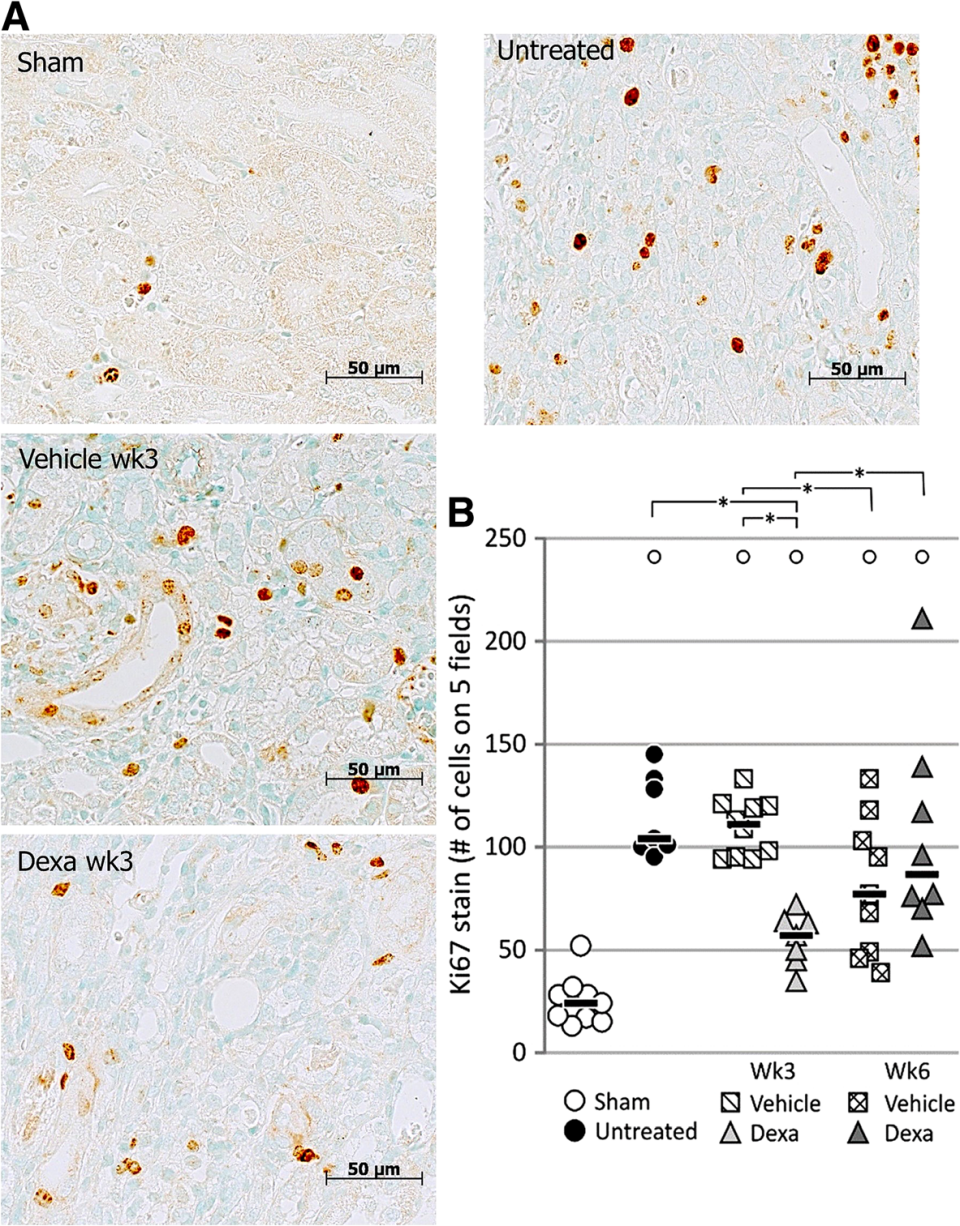
Evaluation of cellular proliferation (Ki67 immunostaining) in the ischemic kidneys. UIRI was performed for 21 min at 36 °C, *n* = 8 in the untreated group, *n* = 10 in sham, dexamethasone and vehicle treatment groups. Animals were euthanized after treatment (3 weeks after UIRI) and 3 weeks after treatment (6 weeks after UIRI). **a**: Representative images of Ki67 immunostained ischemic kidney tissue (magnification: 500x). **b**: Histological quantification of Ki67 positivity in ischemic kidneys. °: *p* < 0.05 vs. Sham, *: *p* < 0.05

Using Ki67 immunostaining, we evaluated the proliferative response in the ischemic kidneys (Fig. 4). Untreated ischemic kidneys showed significant increased proliferation as compared to sham (115 ± 20 vs. 25 ± 12 Ki67 positive cells/field) (*p* < 0.05) 3 weeks after UIRI. A similar amount of Ki67-positive cells were observed in the kidneys of vehicle-treated animals as compared to untreated animals.

**Fig. 5:**
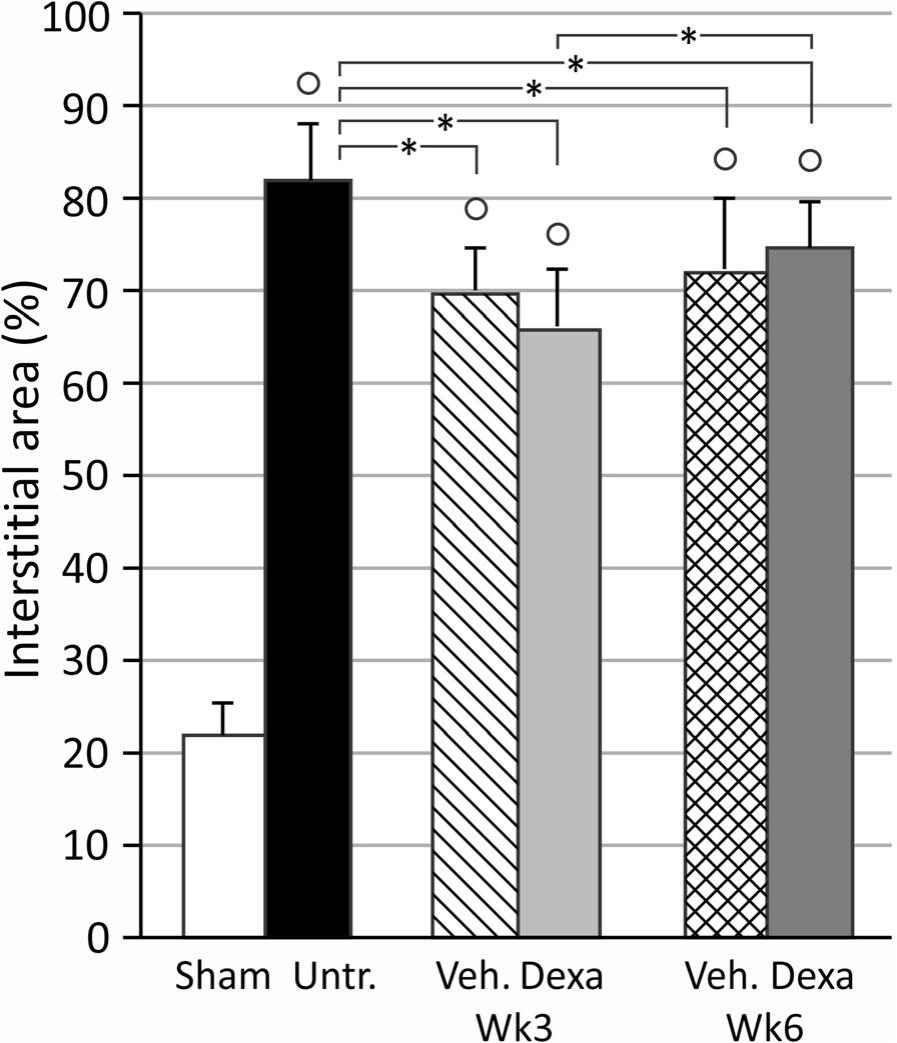
Histological evaluation of tubulointerstitial area in the ischemic kidneys. UIRI was performed for 21 min at 36 °C, *n* = 8 in the untreated group, n = 10 in sham, dexamethasone and vehicle treatment groups. Animals were euthanized after treatment (3 weeks after UIRI) and 3 weeks after treatment (6 weeks after UIRI). °: *p* < 0.05 vs. Sham, *: *p* < 0.05

Renal UIRI without contralateral nephrectomy induced a significant expansion of the tubulointerstitial area in untreated animals as compared to shams (82.0 ± 6.2% vs. 21.9 ± 3.3%) (*p* < 0.05) at week 3. In vehicle treated animals tubulointerstitial expansion was still prominent although slightly lower as compared to untreated animals (69.9 ± 4.6% vs. 82.0 ± 6.2%) (*p* < 0.05) (Fig. 5).

**Fig. 6:**
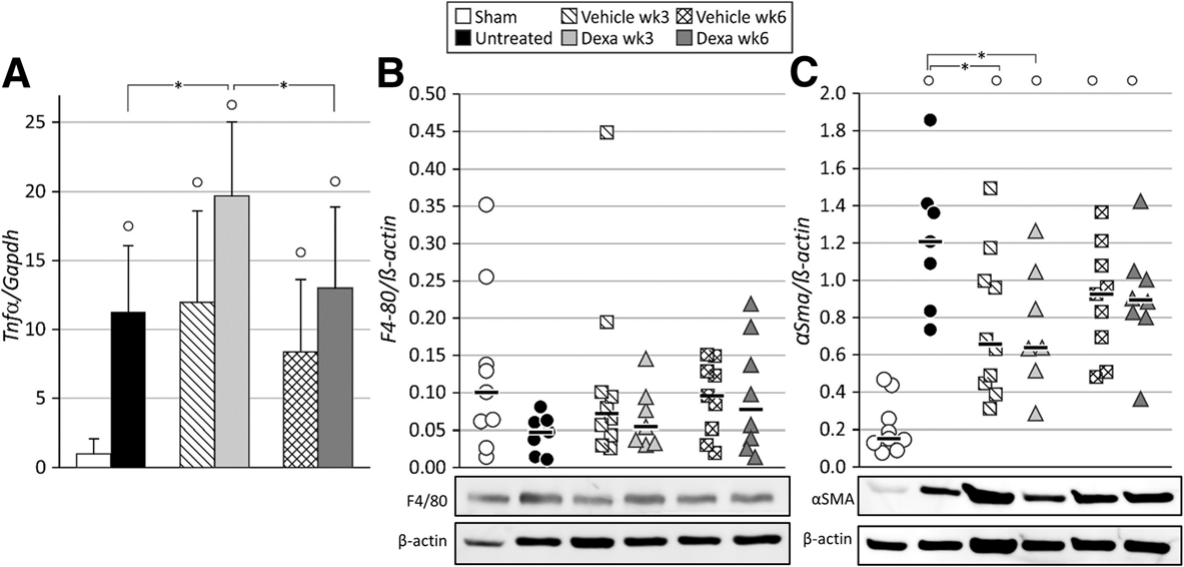
Evaluation of inflammatory and fibroblast markers in the ischemic kidneys. UIRI was performed for 21 min at 36 °C, *n* = 8 in the untreated group, *n* = 10 in sham, dexamethasone and vehicle treatment groups. Animals were euthanized after treatment (3 weeks after UIRI) and 3 weeks after treatment (6 weeks after UIRI). **a**: TNFα gene expression (qPCR). **b**: F4/80 macrophage/monocyte protein expression with representative Western blots for F4/80/β-actin expression. **c**: α-SMA fibroblast protein expression with representative Western blots for α-SMA/β-actin expression. °: *p* < 0.05 vs. Sham, *: *p* < 0.05

Furthermore, the amount of the F4/80 glycoprotein and α-SMAprotein, expressed by monocytes/macrophages and myofibroblasts, respectively, was quantified by Western blot. Similar amounts of F4/80 protein were detected in ischemic kidneys of untreated or vehicle treated animals, 3 weeks after UIRI (Fig. 6b). As shown in Fig. 6c, there is a significant increased α-SMA protein expression in the ischemic kidney of untreated animals as compared to sham (1.21 ± 0.38 ng vs. 0.21 ± 0.14 ng). Vehicle-treated animals showed decreased α-SMA protein expression as compared to untreated animals 3 weeks after UIRI (0.76 ± 0.39 ng vs. 1.21 ± 0.38 ng), but still significantly higher than sham (*p* < 0.05). In addition, expression of α-SMA and, to a modest extent, F4/80 showed a tendency to increase from week 3 to week 6 after UIRI both in vehicle and dexamethasone treated groups (Fig. 6c). Representative images of the histological quantification of F4/80 and α-SMA expression are presented in the supplementary data (Additional file 1: Figure S1 and Additional file 2: Figure S2).

### Temporary dexamethasone treatment attenuates development of fibrosis after ischemic AKI

Animals received dexamethasone treatment during 3 weeks at a dose of 10 mg/kg/day, after which they were euthanized. No noticeable adverse health effects were observed upon dexamethasone treatment. The renal atrophy inherent to the UIRI model was not prevented or attenuated by dexamethasone treatment (Fig. 1). However, the contralateral kidneys were significantly less hypertrophied as compared to the vehicle group (6.9 ± 0.4 mg/g vs. 7.4 ± 0.4 mg/g) (*p* < 0.05) and did not significantly differ from the sham group (Fig. 1). This effect is driven by kidney weight only (not body weight) as shown in Additional file 3: Figure S3.

Expression of the pro-fibrotic genes *collagen I* and *Ccn2* was significantly lower after dexamethasone-treatment as compared to vehicle treatment (11.6 ± 1.3 vs. 17.1 ± 4.4 fold and 2.0 ± 0.3 vs. 2.7 ± 0.9 fold resp.) (*p* < 0.05) (Figs. 2c and 3b). The decrease in *collagen I* gene expression upon dexamethasone treatment was confirmed by collagen I immunostaining (Fig. 2a and b), where significantly less deposition was present in the dexamethasone treated ischemic kidney as compared to untreated animals (4.5 ± 1.9 vs. 7.6 ± 2.7%) (*p* < 0.05) but not to vehicle-treated animals. Also, after 3 weeks of dexamethasone treatment, gene expression of *Pai-1* and *Ccn3* increased further as compared to the untreated group (29.3 ± 7.8 vs. 20.5 ± 4.1-fold and 15.9 ± 2.0 vs. 10.1 ± 4.6-fold resp.) (*p* < 0.05), but not as compared to the vehicle-treated group (Fig. 3c and d). Of these, only the anti-fibrotic *Ccn3* gene expression tended towards higher expression upon dexamethasone treatment as compared to vehicle treatment (*p* = 0.097) (Fig. 3d). There was no difference in the expression of *Tgfβ* after dexamethasone treatment as compared to vehicle treatment (Fig. 3a).

Dexamethasone treatment induced a significant decrease of tubulointerstitial area as compared to untreated animals (66.0 ± 6.4% vs. 82.0 ± 6.2%) (*p* < 0.05), but not vehicle treated animals (Fig. 5).

The expression of the pro-inflammatory cytokine *Tnfα* was significantly increased after dexamethasone treatment as compared to untreated animals (19.7 ± 5.3 vs. 11.3 ± 4.8-fold) (*p* < 0.05), but not vehicle-treated animals (Fig. 6a). Dexamethasone treatment did not have an effect on the amount of F4/80 protein in the ischemic kidney (Fig. 6b, Additional file 4: Figure S4a), however it induced a significant decrease in α-SMA protein expression in the ischemic kidney as compared to the untreated group (0.75 ± 0.33 ng vs. 1.21 ± 0.38 ng) (*p* < 0.05). No difference was seen when compared to vehicle treatment (Fig. 6c). Upon dexamethasone treatment, significantly less cell proliferation can be observed in the ischemic kidney as compared to vehicle treatment (56 ± 16 vs. 110 ± 14 Ki67 positive cells/field) and no treatment (56 ± 16 vs. 105 ± 20 Ki67 positive cells/field) (*p* < 0.05) (Fig. 4b).

### Temporary dexamethasone treatment does not appear to permanently attenuate renal decay after ischemic AKI

After the additional 3-week follow-up, renal atrophy further aggravated both in vehicle- and dexamethasone-treated animals, with a nearly identical reduction in renal mass of 45% to ca. 65% of control kidney weight. The contralateral kidney of dexamethasone-treated animals demonstrated increased hypertrophy at this time-point, to similar levels as vehicle-treated animals (Fig. 1).

The expression of the fibrosis-related genes *collagen I* and *Ccn2* remained stably upregulated in vehicle-treated animals after the follow-up period (Figs. 2c and 3b). In dexamethasone-treated animals, *collagen I* and *Ccn2* gene expression was significantly increased (15.1 ± 2.8 vs. 11.6 ± 1.3 and 3.9 ± 2.0 vs. 2.0 ± 0.3-fold resp.) (*p* < 0.05) to match the expression of vehicle-treated animals (Figs. 2c and 3b). Quantification of collagen I immunostaining showed increased collagen I deposition in the ischemic kidneys of vehicle-treated animals (7.7 ± 3.4% vs. 5.1 ± 1.5%) (*p* < 0.05), whereas collagen I deposition in ischemic kidneys of dexamethasone-treated animals was not significantly increased as compared to immediately after treatment (Fig. 2b). After the follow-up period, gene expression of *Pai-1* and *Tgfβ* was decreased both in vehicle and dexamethasone treated groups as compared to immediately after treatment (Fig. 3a and c). Gene expression of *Ccn3* remained stably upregulated in both vehicle- and dexamethasone-treated animals.

Tubulointerstitial area remained stably increased in vehicle treated animals at week 6. There is a significant increase of tubulointerstitial area in dexamethasone treated animals at week 6 compared to week 3 (74.8 ± 4.9% vs 66.0 ± 6.4%) (*p* < 0.05) (Fig. 5).

The expression of the pro-inflammatory *Tnfα* remained stably upregulated in vehicle-treated animals after the follow-up period. However, its gene expression was decreased in dexamethasone-treated animals at this time-point as compared to immediately after treatment (13.0 ± 5.9 vs. 19.7 ± 5.3-fold) (*p* < 0.05) to similar levels as vehicle-treated animals (Fig. 6a). Protein expression of F4/80 and α-SMA remained stably upregulated in both vehicle- and dexamethasone-treated animals (Fig. 6b and c). The amount of proliferating cells in the ischemic kidneys of vehicle-treated animals was decreased after the additional follow-up period as compared to immediately after treatment (81 ± 33 vs. 110 ± 14 Ki67 positive cells/field) (*p* < 0.05). However, the amount of proliferating cells in the ischemic kidney of dexamethasone-treated animals was increased at this time-point as compared to immediately after treatment (105 ± 51 vs. 55 ± 13 Ki67 positive cells/field) (*p* < 0.05) to similar amounts as present in vehicle-treated animals (Fig. 4b). Representative images of the histological Ki67 staining at the 6 week time point are presented in the supplementary data (Additional file 5: Figure S5).

## Discussion

It is becoming increasingly clear that incomplete recovery from severe AKI is an important pathway to persistent and progressive CKD with underlying fibrosis. A recent meta-analysis reported that patients surviving an episode of AKI have an 8.8-fold increased risk for CKD and a 3.3-fold increased risk for ESRD [3]. Understanding the mechanisms underlying the progression from acute-to-chronic renal injury is the focus of recent research in the field [15]. Since renal fibrosis is nearly always preceded by and closely associated with inflammation, both in patients [16] and experimental models of fibrosis [17], it is thought to be one of the major processes that contributes to progression of renal disease [18]. Also, experimental studies demonstrated that even when renal function recovers after AKI, pro-inflammatory and pro-fibrotic pathways remain active [19]. Several laboratories demonstrated that suppression of the inflammatory response can reduce post-ischemic injury 24–48 h after the ischemic insult [20]. Yet, few studies investigated the long-term effects of inflammatory modulation on development of CKD, which, in the context of the recently appreciated AKI-to-CKD link, is of major therapeutic interest. Therefore, we here evaluated whether temporary treatment (3 weeks) with immune-suppressive dexamethasone is able to attenuate the development of post-ischemic renal fibrosis and avert the progression from acute to chronic renal injury.

In the current study, renal atrophy is pronounced and progressive, with loss of renal mass up to 44 and 64% within 3 and 6 weeks after the ischemic insult, respectively (Fig. 1). Treatment with dexamethasone was unable to attenuate or prevent this loss of renal mass. However, adaptive growth of the contralateral kidney to compensate for the loss of functional renal tissue [21] of the ischemic kidney did not occur immediately after dexamethasone treatment. This could indicate that dexamethasone was able to rescue a certain degree of renal function (however not renal mass) of the ischemic kidney. Although the treatment regimen and dosing was the same as in the experiment of Zager et al. (2011), who observed 50% loss of renal mass in dexamethasone treated animals as compared to 66% in untreated animals [12] we can only speculate as to why dexamethasone did not influence renal atrophy in our study. Most likely this is due to differences in the severity of the ischemic insult, which is very subjective to inter-laboratory variation as well as mouse strain dependent susceptibility to ischemic AKI [22]. The presumption that the loss of function of the ischemic kidney was attenuated by dexamethasone treatment, is further supported by the mitigated development of post-ischemic fibrosis, as evidenced by a reduction in collagen I gene- and protein expression, and *Ccn2* gene expression (Figs. 2b-c and 3b). Although we did not assess the effect of dexamethasone treatment on collagen I expression of fibroblasts within the kidney ex vivo or in vitro, among others, Zern et al. [23] and Waqar et al. [24] previously reported that treatment of fibroblasts with dexamethasone lead to decreased collagen I and collagen IV expression accompanied by decreased collagen synthesis in vitro. Moreover, gene expression of *Ccn3*, that has been shown to have anti-fibrotic properties [25], was significantly elevated upon dexamethasone treatment (Fig. 3d). Furthermore, although gene expression of the pro-fibrotic *Pai-1*, an inhibitor of matrix degradation, appeared to be significantly elevated after dexamethasone treatment in comparison to the untreated condition, it did not differ from the vehicle-treated group. Similar results were observed in mercury chloride-induced nephropathy [26]. Despite these molecular observations, dexamethasone treatment was not able to attenuate expansion of the tubulointerstitial area after UIRI.

To further examine the effect of dexamethasone treatment on the fibrotic response after an acute ischemic insult, protein expression of α-SMA, a marker for activated fibroblasts, was determined. In normal kidney tissue, α-SMA staining is only found in smooth muscle cells, mostly in blood vessels [27]. In fibrotic diseases, α-SMA expression of myofibroblasts is recognized as a hallmark of their emergence and an indicator of disease severity [28]. In our study, dexamethasone did not have an effect on the degree of α-SMA expression as compared to vehicle-treatment. In this respect it is worthwhile to note that α-SMA in myofibroblasts appears to have a suppressing role in tissue fibrosis progression, and forced expression in α-Sma^−/−^animals ameliorates fibrosis in the model of ureter obstruction and mesangioproliferative glomerulonephritis [28].

In addition to fibrosis, the post-ischemic period is characterized by an active inflammatory response, resulting from both activation of resident inflammatory cells and recruitment of circulating inflammatory cells [16]. Baeck et al. (2015) have shown, by means of a fluorescent double stain (Ki67 and F4/80), that significant proliferation of monocyte-derived macrophages occurs in the ischemic kidney, both in the acute (day 3, day 5) and early chronic phase (day 20) [29], thereby amplifying and prolonging the local inflammatory response [30]. In accordance with this, we observed significant infiltration of inflammatory cells in the ischemic kidney, quantified by protein expression of the F4/80 glycoprotein, which is expressed by murine monocytes/macrophages. Also, significantly elevated gene expression of the inflammatory cytokines *Tnfα* and *Tgfβ* supports ongoing post-ischemic inflammation. Increased gene expression of the inflammatory *Tnfα* was observed in the ischemic kidney after dexamethasone treatment as compared to untreated animals. Although unexpected at first sight, it has been shown that *Tnfα* can modulate the expression of the glucocorticoid receptor isoforms in such a manner that glucocorticoid resistance may occur [31]. Consistent with this is the fact that, steroid insensitivity has been described in renal epithelial cells [32] and macrophages in the lung [33]. Thus, it might not be surprising that dexamethasone treatment did not have an effect on the amount of F4/80 macrophage protein in the ischemic kidney. Also, it was shown by Castano et al. (2009) that it is possible to achieve attenuation of fibrosis without affecting the number of interstitial macrophages, quantified by F4/80 protein expression, which is also in line with our observations [34].

On a technical side, the discrepancy we observed between Western blot and immunohistological quantitation of α-SMA and F4/80 expression is puzzling. A potential explanation could be that our tissue sampling strategy consistently lead to the use of tissue fragments that were harvested from the poles of the kidneys for Western blot whereas immunohistology was performed on complete transversal sections. Consequently, our Western blot samples could have contained less material originating from the inner cortex and outer medullary region which are the most affected by ischemia/reperfusion.

In normal physiological conditions, renal tubular cells have a low proliferation rate. The current consensus on renal tubular regeneration states that restoration of the tubular epithelium after an acute injury occurs predominately via proliferation of surviving epithelial cells that undergo dedifferentiation, primarily within the first 2 weeks [35]. In our study, a significant increase in cell proliferation in the injured kidney is seen up to 6 weeks after UIRI. Since successful proliferation of proximal tubule cells, i.e. no G2/M cell cycle arrest, is associated with attenuation of fibrosis [36], it was quite unexpected that temporary attenuation of renal decay immediately after dexamethasone treatment went along with an overall decrease in cell proliferation in the ischemic kidney. It should be noted, however, that quantification of proliferation in our study made no distinction between the renal cell types (epithelial cells, fibroblasts, inflammatory cells), nor cell cycle phase or their location (i.e. tubules vs. interstitium) due to severe distortion of the physiological tubulo-interstitial structure. However, independent studies reported decreased infiltration of lymphocytes [26], T-cells [37] and dendritic cells [37] upon dexamethasone treatment. The question which proliferative cell type and cell cycle phase was primarily affected by dexamethasone treatment in the current setting lies outside the scope of the current report. However, we observed that a subset of interstitial cells were also positive for Ki67.

As a three-week dexamethasone treatment regimen attenuated the development of renal fibrosis after UIRI, we included an additional three weeks of follow-up without treatment, to evaluate whether the beneficial effects of dexamethasone treatment persist during the further course of the ischemic renal pathology. As compared to immediately after end of treatment, a further loss of renal mass was observed, indicating that the progressive nature of the pathological course could not be attenuated by dexamethasone. The fact that compensatory hypertrophy of the contralateral kidney emerged after the three-week follow-up period indicates that the positive effect of dexamethasone is temporary, and that longer or continuous treatment is necessary to reach persistent benefit from treatment. After the follow-up period, expression of the pro-fibrotic genes *collagen I* and *Ccn2* was significantly increased as compared to the end-of-treatment time point (Figs. 2c and 3b), indicating that the beneficial effect of dexamethasone-treatment also on pro-fibrotic gene expression is transient. However, analysis of collagen I deposition by immunostaining showed no further progression of fibrosis during the follow-up period in the dexamethasone group, whereas vehicle treated animals displayed progressive renal fibrosis and increasing collagen I staining in the ischemic kidney (Fig. 2b).

Although the results of these experiments are consistent with respect to the long term effect of temporary immunosuppression, we must acknowledge some limitations to this study. Firstly, no functional assessment in serum or urine samples was included since the study setup was focussed on the effects on renal pathology. Secondly, as mentioned earlier, no distinction was made between the renal cell types, cell cycle phase or location of proliferating Ki67^+^-cells due to severe distortion of the physiological tubule-interstitial structure. Third, we observed for some parameters that vehicle treatment can induce similar effects as dexamethasone. Although we can only speculate on the reason for this, it emphasizes that comparative intervention studies cannot rely on untreated groups alone and should include vehicle treated groups to study the true potential of a particular compound.

## Conclusions

In conclusion, dexamethasone treatment attenuates the development of renal fibrosis after an acute ischemic event, as evidenced by decreased upregulation of *collagen I* and *Ccn2* gene expression and decreased collagen I immunostaining immediately after a 3-week treatment period. Dexamethasone did not exert an effect on macrophage F4/80 expression, and tended to increase *Tnfα* gene expression. Additional follow-up of the dexamethasone-treated animals indicated that temporary dexamethasone treatment does not appear to permanently attenuate upregulation of fibrosis-related genes, whereas collagen I protein deposition tends to be affected longer. Overall immune suppressive treatment strategies can attenuate the development of CKD/fibrosis after an acute ischemic event, and can provide a basis for a multi-factorial treatment strategy. However, persistent treatment until near complete resolution of inflammation might be required.

## Additional files

Additional file 1 **Figure S1.** Histological evaluation of inflammatory marker in the ischemic kidneys. UIRI was performed for 21 min at 36 °C, *n* = 8 in the untreated group, *n* = 10 in sham, dexamethasone and vehicle treatment groups. Animals were euthanized after treatment (3 weeks after UIRI) and 3 weeks after treatment (6 weeks after UIRI). **A:** F4–80 macrophage/monocyte area % stain**. B**: Representative images of F4–80 immunostained ischemic kidney tissue (magnification: 500x). °: *p* < 0.05 vs. Sham, *: *p* < 0.05. (JPG 1991 kb)

Additional file 2 **Figure S2.** Histological evaluation of fibroblast marker in the ischemic kidneys. UIRI was performed for 21 min at 36 °C, *n* = 8 in the untreated group, *n* = 10 in sham, dexamethasone and vehicle treatment groups. Animals were euthanized after treatment (3 weeks after UIRI) and 3 weeks after treatment (6 weeks after UIRI). **A:** α-SMA fibroblast area % stain**. B**: Representative images of α-SMA immunostained ischemic kidney tissue (magnification: 50x). °: *p* < 0.05 vs. Sham, *: *p* < 0.05. (JPG 1123 kb)

Additional file 3 **Figure S3.** Body weight and mass of the ischemic and contralateral kidneys at euthanasia. UIRI was performed for 21 min at 36 °C, *n* = 8 in the untreated group, *n* = 10 in sham, dexamethasone and vehicle treatment groups. Animals were euthanized after treatment (3 weeks after UIRI) and 3 weeks after treatment (6 weeks after UIRI). **A:** Body weight of animals at euthanasia. **B:** Mass of ischemic and contralateral kidneys at euthanasia. °: *p* < 0.05 vs. Sham, *: *p* < 0.05. (JPG 919 kb)

Additional file 4 **Figure S4.** Original blots of F4/80 and α-SMA protein expression. Dex/Veh Wk3: Animals were euthanized after treatment with dexamethasone/vehicle respectively (3 weeks after UIRI); Dex/Veh Wk6: animals were euthanized 3 weeks after treatment with dexamethasone/vehicle respectively (6 weeks after UIRI); Untreated: animals were euthanized 3 weeks after UIRI; Sham: animals were euthanized 6 weeks after mock-UIRI. A: Original Western Blot of F4/80 macrophage/monocyte and β-actin protein expression. It should be noted that under non-reducing circumstances the molecular weight of F4/80 is 102 kDa. For reducing circumstances (i.e. boiling in 2-mercaptoethanol), as used in our study, Starkey et al. [38] reported that the antigen is cleaved in two fragments of 20 kDa and 80 kDa, the latter of which detected in our analysis. B: Original Western Blot of α-SMA macrophage/monocyte and β-actin protein expression. (JPG 439 kb)

Additional file 5 **Figure S5.** Evaluation of cellular proliferation (Ki67 immunostaining) in the ischemic kidneys at week 6. UIRI was performed for 21 min at 36 °C, *n* = 8 in the untreated group, *n* = 10 in sham, dexamethasone and vehicle treatment groups. Animals were euthanized after treatment (3 weeks after UIRI) and 3 weeks after treatment (6 weeks after UIRI). Representative images of Ki67 immunostained ischemic kidney tissue (magnification: 500x). (JPG 1000 kb)

## Abbreviations

AKI: Acute Kidney Injury
CKD: Chronic Kidney Disease
ESRD: End Stage Renal Disease
UIRI: Unilateral Ischemia Reperfusion Injury
