# Supplementary figures and images for: Short-term dexamethasone treatment transiently, but not permanently, attenuates fibrosis after acute-to-chronic kidney injury
Source: BMC Nephrol. 2018 Dec 3;19:343. doi: 10.1186/s12882-018-1151-7 (PMC6276259; doi:10.1186/s12882-018-1151-7)

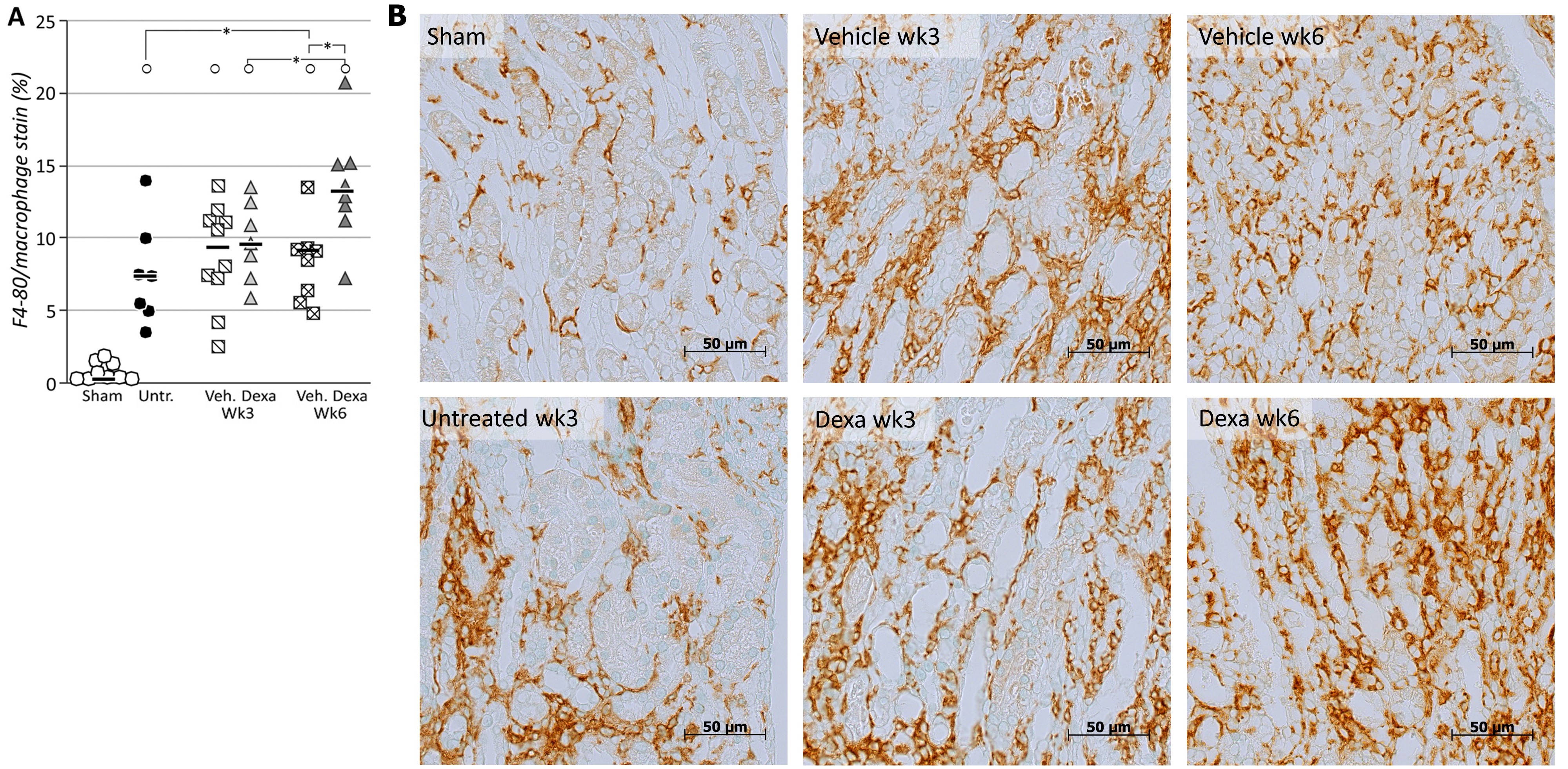

Supplement: Supplementary file 1 — Figure S1. Histological evaluation of inflammatory marker in the ischemic kidneys. UIRI was performed for 21 min at 36 °C, n = 8 in the untreated group, n = 10 in sham, dexamethasone and vehicle treatment groups. Animals were euthanized after treatment (3 weeks after UIRI) and 3 weeks after treatment (6 weeks after UIRI). A: F4–80 macrophage/monocyte area % stain. B: Representative images of F4–80 immunostained ischemic kidney tissue (magnification: 500x). °: p < 0.05 vs. Sham, *: p < 0.05. (JPG 1991 kb) [file 12882_2018_1151_MOESM1_ESM.jpg]

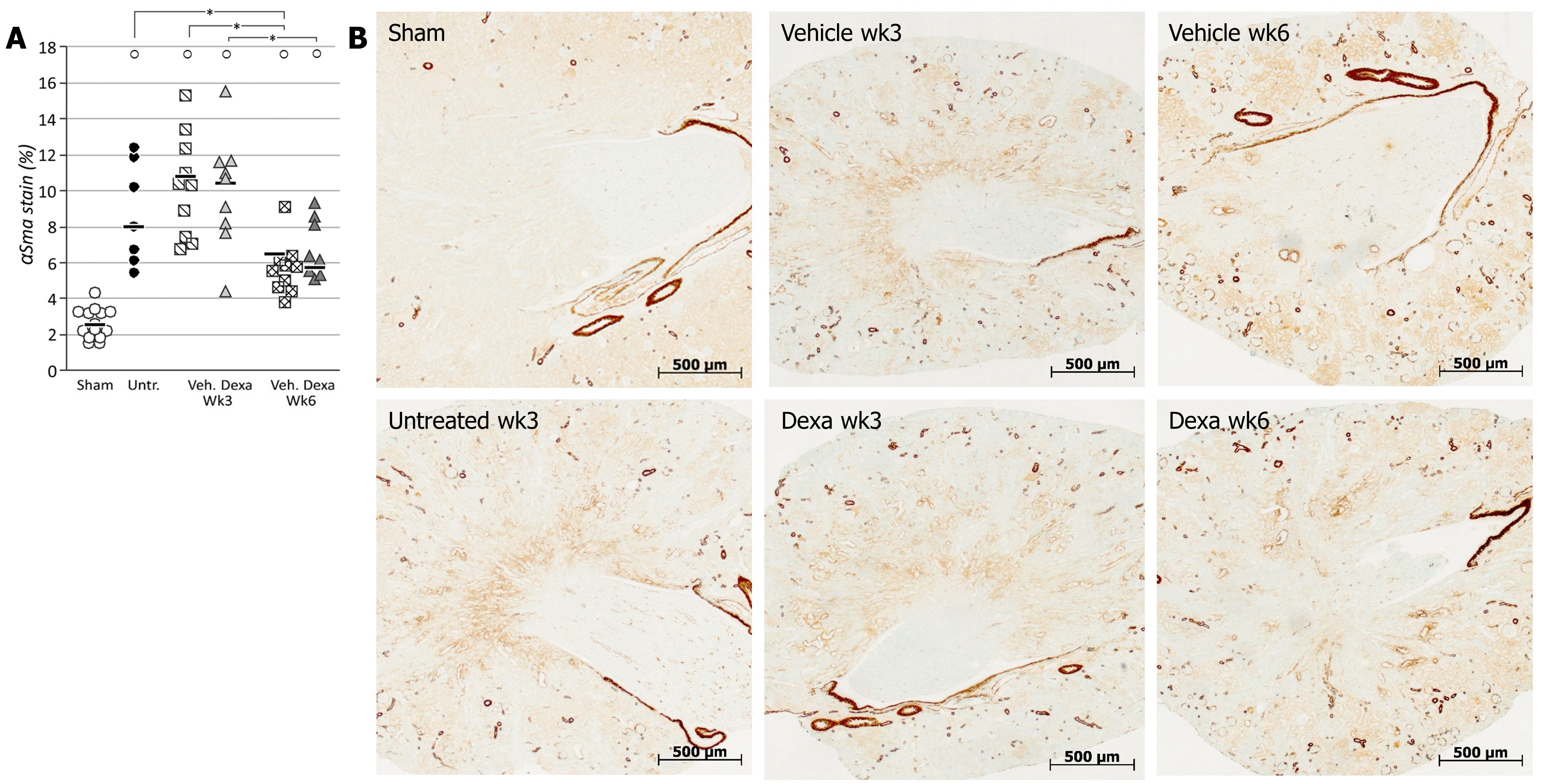

Supplement: Supplementary file 2 — Figure S2. Histological evaluation of fibroblast marker in the ischemic kidneys. UIRI was performed for 21 min at 36 °C, n = 8 in the untreated group, n = 10 in sham, dexamethasone and vehicle treatment groups. Animals were euthanized after treatment (3 weeks after UIRI) and 3 weeks after treatment (6 weeks after UIRI). A: α-SMA fibroblast area % stain. B: Representative images of α-SMA immunostained ischemic kidney tissue (magnification: 50x). °: p < 0.05 vs. Sham, *: p < 0.05. (JPG 1123 kb) [file 12882_2018_1151_MOESM2_ESM.jpg]

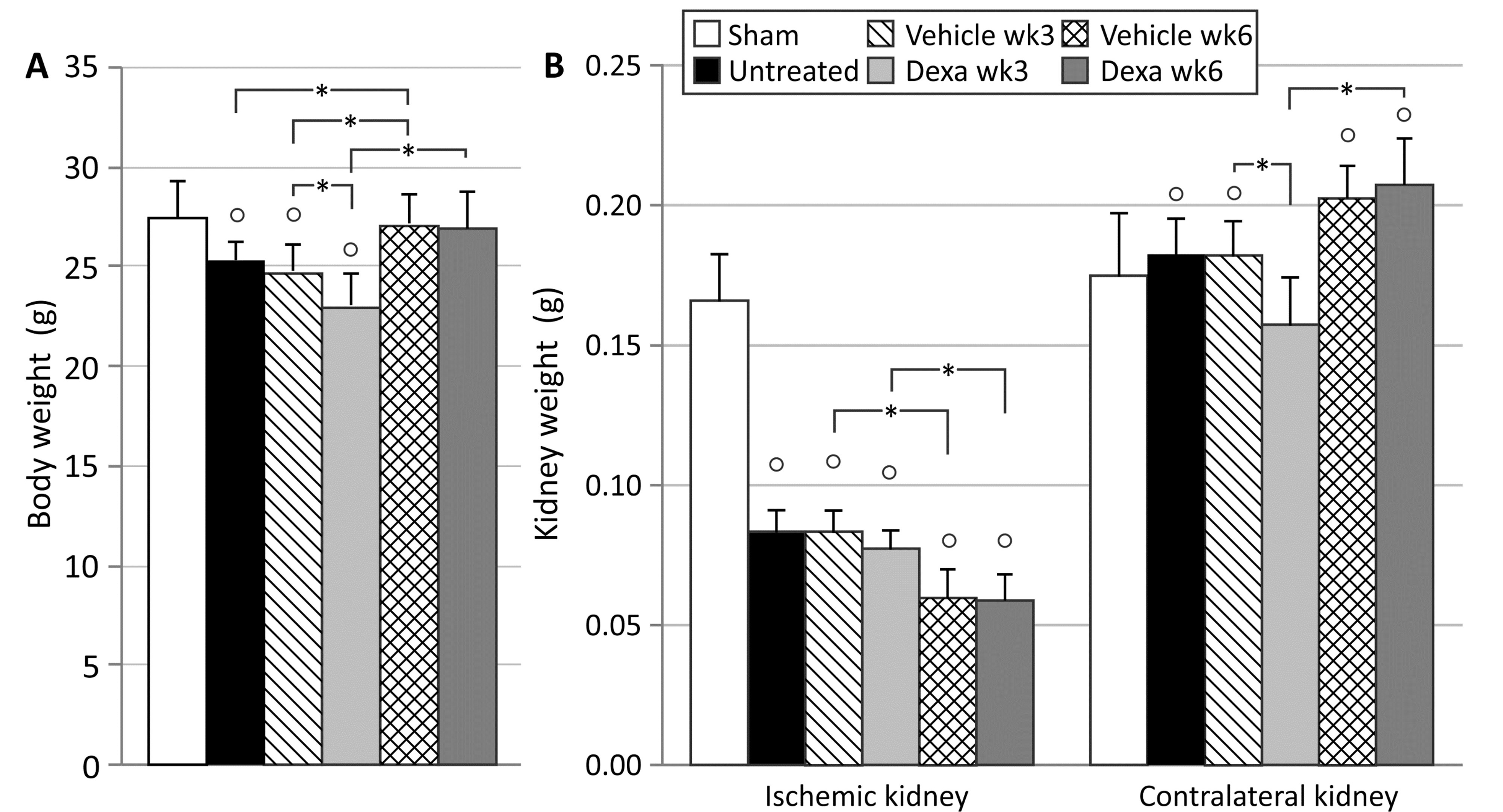

Supplement: Supplementary file 3 — Figure S3. Body weight and mass of the ischemic and contralateral kidneys at euthanasia. UIRI was performed for 21 min at 36 °C, n = 8 in the untreated group, n = 10 in sham, dexamethasone and vehicle treatment groups. Animals were euthanized after treatment (3 weeks after UIRI) and 3 weeks after treatment (6 weeks after UIRI). A: Body weight of animals at euthanasia. B: Mass of ischemic and contralateral kidneys at euthanasia. °: p < 0.05 vs. Sham, *: p < 0.05. (JPG 919 kb) [file 12882_2018_1151_MOESM3_ESM.jpg]

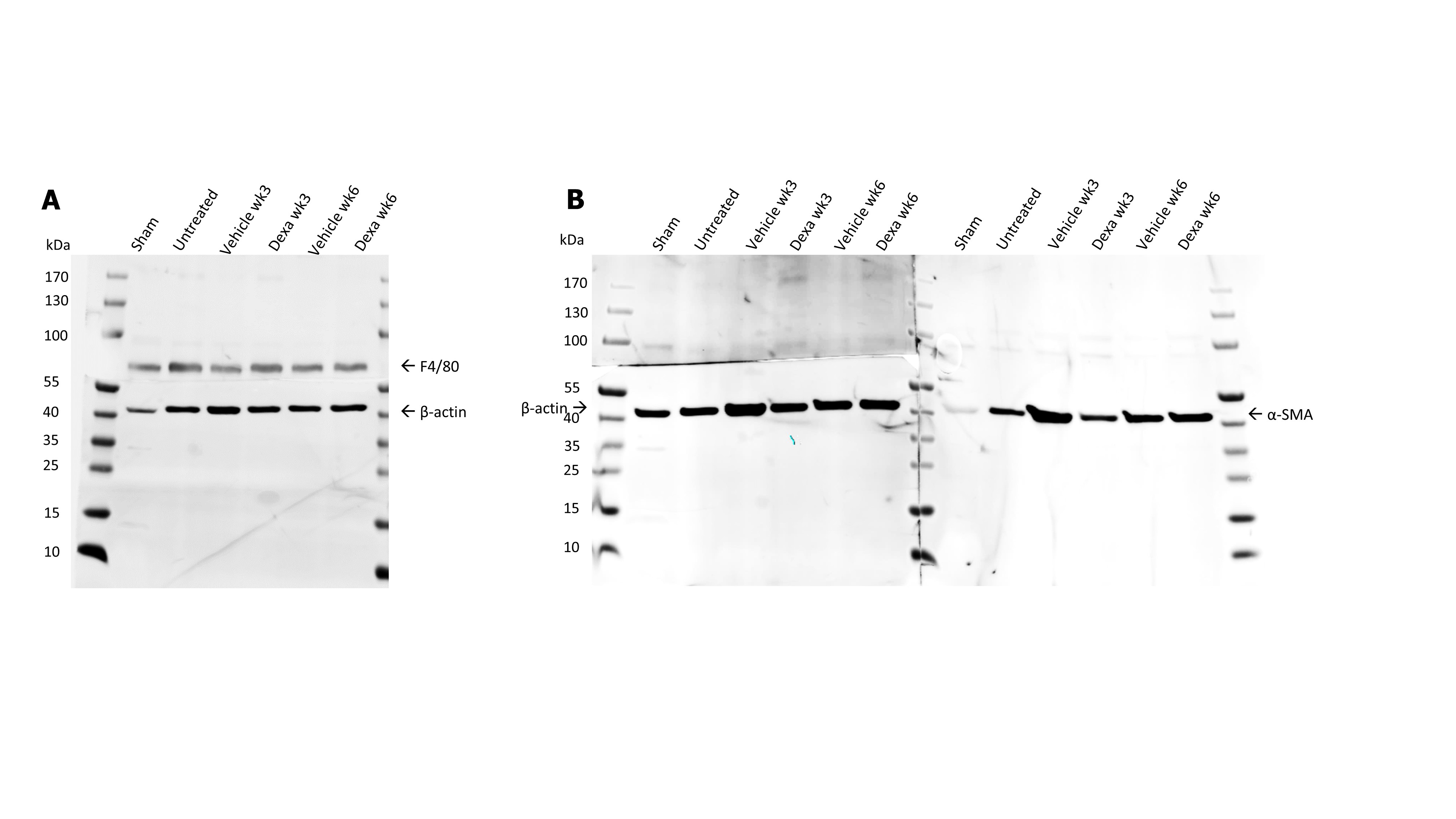

Supplement: Supplementary file 4 — Figure S4. Original blots of F4/80 and α-SMA protein expression. Dex/Veh Wk3: Animals were euthanized after treatment with dexamethasone/vehicle respectively (3 weeks after UIRI); Dex/Veh Wk6: animals were euthanized 3 weeks after treatment with dexamethasone/vehicle respectively (6 weeks after UIRI); Untreated: animals were euthanized 3 weeks after UIRI; Sham: animals were euthanized 6 weeks after mock-UIRI. A: Original Western Blot of F4/80 macrophage/monocyte and β-actin protein expression. It should be noted that under non-reducing circumstances the molecular weight of F4/80 is 102 kDa. For reducing circumstances (i.e. boiling in 2-mercaptoethanol), as used in our study, Starkey et al. [38] reported that the antigen is cleaved in two fragments of 20 kDa and 80 kDa, the latter of which detected in our analysis. B: Original Western Blot of α-SMA macrophage/monocyte and β-actin protein expression. (JPG 439 kb) [file 12882_2018_1151_MOESM4_ESM.jpg]

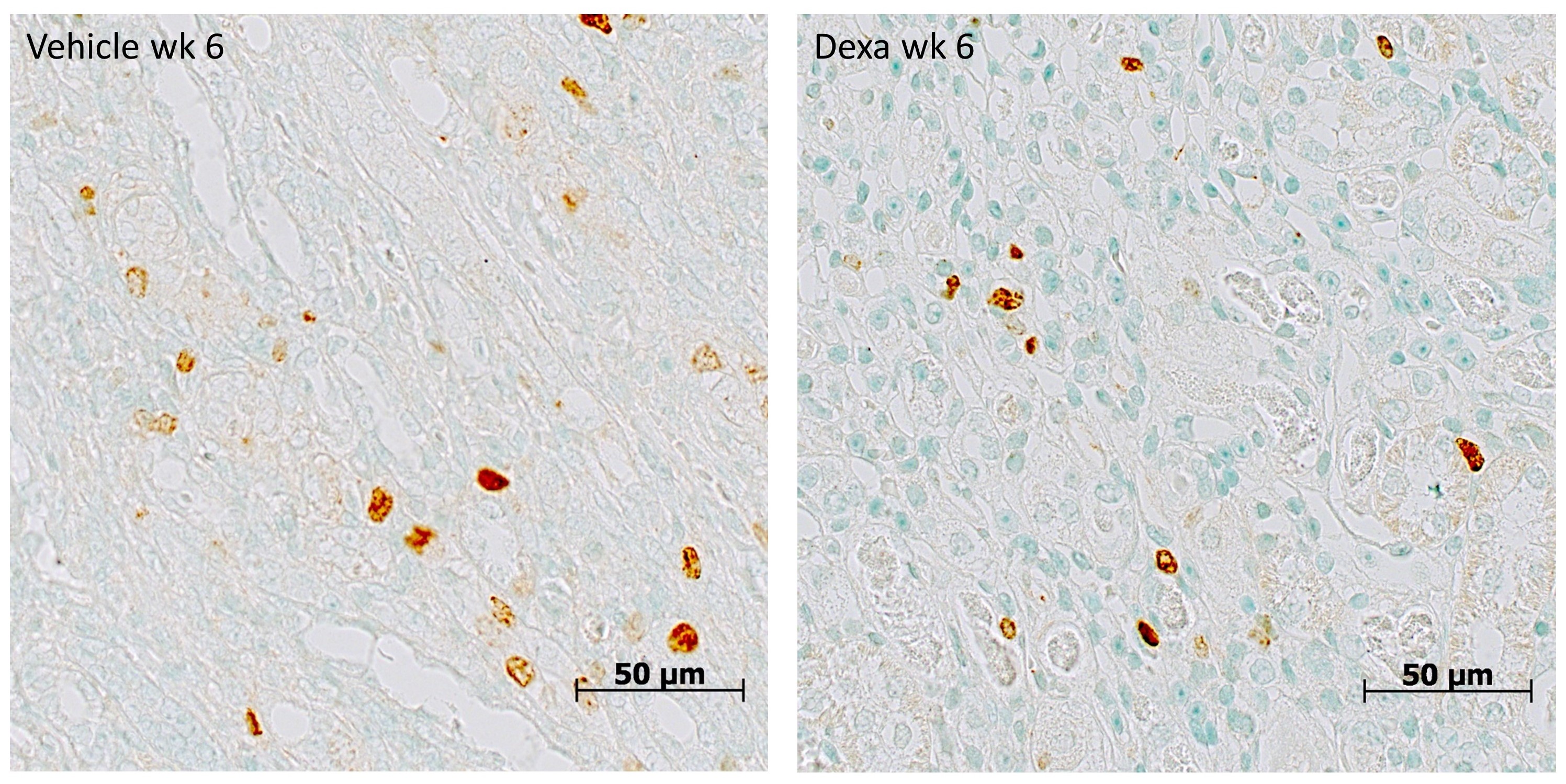

Supplement: Supplementary file 5 — Figure S5. Evaluation of cellular proliferation (Ki67 immunostaining) in the ischemic kidneys at week 6. UIRI was performed for 21 min at 36 °C, n = 8 in the untreated group, n = 10 in sham, dexamethasone and vehicle treatment groups. Animals were euthanized after treatment (3 weeks after UIRI) and 3 weeks after treatment (6 weeks after UIRI). Representative images of Ki67 immunostained ischemic kidney tissue (magnification: 500x). (JPG 1000 kb) [file 12882_2018_1151_MOESM5_ESM.jpg]
